# Biomarker-Driven Pharmacokinetics and Efficacy of Polymyxin B in Critically Ill Patients with XDR-GN Pneumonia

**DOI:** 10.3390/ph18040586

**Published:** 2025-04-17

**Authors:** Wei Zuo, Qianlin Wang, Longxiang Su, Jiaxin Yu, Hongwei Fan, Qiang Fu, Yun Long, Bo Zhang

**Affiliations:** 1 Department of Pharmacy, Peking Union Medical College Hospital, Chinese Academy of Medical Sciences, Beijing 100730, China; eileenzuo@163.com (W.Z.);; 2 State Key Laboratory of Complex Severe and Rare Diseases, Peking Union Medical College Hospital, Chinese Academy of Medical Sciences, Beijing 100730, China; 3 Department of Critical Care Medicine, Peking Union Medical College Hospital, Chinese Academy of Medical Sciences, Beijing 100730, China; 4 Department of Infectious Disease, Peking Union Medical College Hospital, Chinese Academy of Medical Sciences, Beijing 100730, China

**Keywords:** polymyxin B, pharmacokinetic and pharmacodynamics, bronchoalveolar lavage fluid, inflammatory factors

## Abstract

**Background:** Achieving pharmacokinetic/pharmacodynamic (PK/PD) targets is critical for improving treatment success, particularly in critically ill patients. This study investigates the role of inflammatory biomarkers and their influence on the PK/PD characteristics of polymyxin B (PMB) in patients with extensively drug-resistant Gram-negative (XDR-GN) bacterial nosocomial pneumonia. **Methods:** Serial blood and/or bronchoalveolar lavage fluid (BALF) samples were collected at specified time points and analyzed for PMB and/or inflammatory biomarkers, including IL-6 and IL-10. Clinical data were also recorded, and their correlations with PK parameters were further analyzed. **Results:** Among the 27 enrolled patients, 22 (81.5%) achieved treatment success. The pharmacokinetic parameters of PMB included a maximum plasma concentration (C_max_) of 8.3 µg/mL, clearance (CL) of 1.55 L/h, volume of distribution (Vd) of 30.44 L, half-life (t_1/2_) of 19.56 h, steady-state area under the plasma concentration–time curve from time 0 to 24 h (AUC_ss,0–24h_) of 110.08 h·µg/mL, and a plasma protein-binding ratio of 85.53%. The AUC_ss,0–24h_ metric was identified as a robust predictor of clinical efficacy, with an optimal cutoff value of 77.27 h·µg/mL. Notably, 48.15% of patients achieved the target AUC_ss,0–24h_ range of 50–100 h·µg/mL, with 76.95% of these patients attaining treatment success. Another 48.15% of patients exceeded this target, and 92.31% of this subgroup achieved treatment success. PMB demonstrated limited pulmonary penetration, with an epithelial lining fluid (ELF)/plasma ratio of 15.69% [16.86, 18.15]. Furthermore, TNF-α and the IL-6/IL-10 ratio were significantly correlated with PMB PK parameters. **Conclusions:** Our and others’ studies suggest heterogeneity of PMB PK parameters in critically ill patients. The majority of critically ill patients achieved or surpassed the recommended PK/PD targets and attained treatment success through intravenous administration of PMB at a simplified fixed dose. However, PMB did not achieve satisfactory pulmonary concentrations, suggesting that its efficacy may involve alternative mechanisms. The modulation of inflammatory responses may play a pivotal role in the treatment of severe infections, highlighting the potential for biomarker-guided therapeutic strategies.

## 1. Introduction

Colistin (colistin methanesulfonate, CMS) and polymyxin B (PMB) are polymyxins that were approved for clinical use in the 1950s worldwide. Colistin is clinically administered as a prodrug, which requires conversion to the active form in vivo. The conversion efficiency exhibits significant individual variation (20–25%). PMB is directly administered in its active form, resulting in more stable plasma concentration profiles. The elimination of colistin is primarily dependent on renal function. Patients with renal impairment are prone to drug accumulation and increased nephrotoxicity risk. PMB is predominantly cleared via non-renal pathways (∼60% hepatobiliary metabolism), associated with a comparatively lower risk of nephrotoxicity. In China, colistin (colistin sulfate), the active ingredient of which is active colistin, was approved by the National Medical Products Administration (NMPA) in 2018. The use of colistin has declined due to nephrotoxicity and neurotoxicity concerns, as well as the emergence of alternatives [1,2,3,4,5,6,7,8]. With the sharp increase in multidrug-resistant (MDR) and extensively drug-resistant (XDR) Gram-negative infections and the challenges they bring, the use of polymyxins (PMs) was reintroduced as a last resort. Although most MDR or XDR Gram-negative bacteria are susceptible to the conventional antibiotic PM [9], resistance has been reported recently [10,11,12]. As the problem of bacterial resistance is becoming increasingly serious, how to rationally use antimicrobial drugs and optimize antimicrobial treatment regimens has become a clinical issue worth considering. An in-depth understanding of the pharmacokinetics/pharmacodynamics (PK/PD) of antibiotics is critical for optimizing their dosage regimens to maximize their efficacy and minimize their toxicity and resistance.

Owing to the lack of data, the International Consensus Guidelines for the Optimal Use of PMs (hereafter referred to as ICGOUP) recommend similar PK/PD targets for PMB as those listed for CMS. With a minimal inhibitory concentration of 2 mg/L (the EUCAST and CLSI breakpoints both aim to classify bacteria as susceptible, intermediate, or resistant based on MIC, guiding precise clinical use of antimicrobial agents; however, differences in methodologies and dosing standards may lead to discordant interpretations for the same bacterial isolate), an area under the 0–24 h concentration–time curve (AUC_ss,0–24h_) target of 50–100 mg·h/L, corresponding to a C_ss,avg_ of 2–4 mg/L, was suggested [13,14]. This should be considered the maximum tolerable exposure of CMS. First, we hypothesize that the current recommended PK/PD indices are not optimal for PMB because there are several clinical pharmacologic differences between CMS and PMB: (1) CMS is administered as an inactive prodrug [15], whereas PMB is available as the active entity [16]. (2) Thus, the plasma concentrations of CMS increase slowly and vary considerably between individuals. Even with a loading dose, several hours may be needed to achieve effective concentrations [17,18]. (3) In contrast to CMS, PMB is cleared via the non-renal pathway; thus, the PK of PMB is not affected by renal function. The risk of acute kidney injury (AKI) appears to be lower with PMB [19,20,21,22,23,24,25,26,27]. (4) PMB may have different toxicodynamic (TD) characteristics than CMS does [14]. For these reasons, the PK/PD targets may differ between colistin and PMB.

Preclinical evidence from lung infection models suggests poor exposure and response after intravenous administration of PM monotherapy [28,29], but clinical evidence is available for its effectiveness [27,30]. What is more, in China, a simplified fixed dose (100 mg loading dose, followed by 50–100 mg q12 h maintenance doses, indicated in the drug instructions), which is lower than the TBW-adjusted dose (2.0–2.5 mg/kg loading dose, followed by 1.25–1.5 mg/kg q12 h and maintenance dose, recommended by ICGOUP), could exert an effective therapeutic role. Together with the clinical experience, we began to think about which factors determine and influence the effect of PMB in treating XDR-GN bacterial nosocomial pneumonia. All sorts of factors might be involved in it, including differences between in vivo and in vitro models (e.g., in critically ill patients, systematic inflammation may attribute to more significant damage to the cellular permeability barrier), measurement technique, the synergistic effect of combination drugs [30,31,32,33], racial difference, and so on. We proposed whether the concentration of PMB in the epithelial lining fluid (ELF) is a proper parameter for predicting and/or assessing clinical efficacy, as well as what its influence factors are.

Although many studies have focused on elucidating the PK/PD profile of PMB, these studies were normally carried out in the context of PMB monotherapy, in animal or in vitro models, or in small samples of healthy volunteers or noncritical patients [34,35,36,37]. Although a growing number of studies have focused on population PK (PPK) [35,38,39], the ability to evaluate the impact of covariates and interoccasion variability on PPK parameters has been restricted by the use of sparse resampling techniques in small samples, especially for critically ill patients, who have significantly more individual variation.

PMB is a valuable last-resort antibiotic for treating MDR Gram-negative infections, particularly when other options are limited (e.g., inaccessible to new drugs). However, its use should be carefully considered due to the potential for nephrotoxicity and the emergence of resistance. There were still gaps in PK/PD knowledge for PMB in real-world clinical settings. To date, research into the PK/PD features of PMB is limited in seriously ill patients with lung infection, and there is a discrepancy between in vitro tissue distribution data and in vivo anti-infection efficacy [27,30,31,32,33]. There is also a lack of real-time assays for concentrations (PK) and biomarkers (PD). Whether certain patient characteristics, such as gender, BMI, renal function, and biochemical indexes, are independent predictors of failure to achieve aggressive PK/PD targets is still unknown. Recent studies have emphasized the significance of multi-biomarker strategies and the feasibility of personalized medicine in infection management. Further verification of biomarkers on a large scale and their integration into clinical practice are advocated to maximize their efficacy in future treatment [40,41,42]. This gap makes it challenging for clinicians to determine the optimal dosing strategies for PMB in a timely manner based on individual patient characteristics, potentially leading to suboptimal treatment outcomes and increased risk of resistance development.

Therefore, we performed this prospective cohort study on the real-world use of PMB to investigate the plasma PK/PD profile of PMB at a simplified dosage and its contributing factors in a cohort of critically ill patients with XDR-GN bacterial nosocomial pneumonia. We also examined the exposure of PMB at the target site by measuring PMB in bronchoalveolar lavage (BAL) samples.

## 2. Results

### 2.1. Clinical Characteristics of the Patients

**Figure 1:**
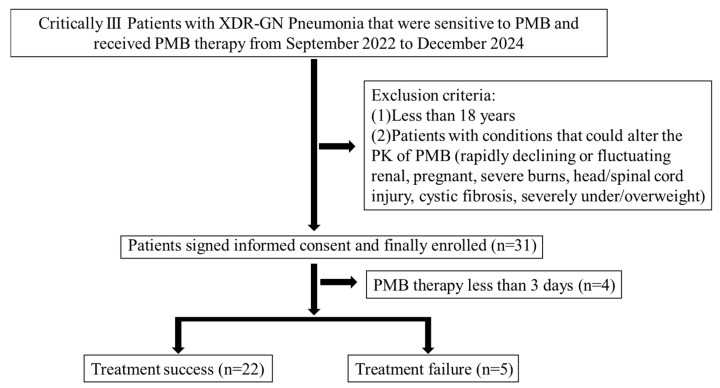
Flowchart of the study inclusion process and groups used in the analysis.

A total of 27 patients who were prescribed PMB for more than 72 h at PUMCH were enrolled in our study (Figure 1), among whom 19 were males. The median age, weight, creatinine clearance rate, APACHE II score, and SOFA score were 68.0 years, 67.5 kg, 72.5 mL/min, 22.0, and 12.0, respectively. Most of the patients had primary diseases, most often cardiovascular diseases. A total of 5 patients experienced treatment failure, while the other 22 had treatment success. Other demographic details of these patients are shown in Table 1.

Patients in the treatment failure group were more likely to have higher APACHE II scores, longer PMB therapy times, and a trend toward a higher percentage of bloodstream infections than patients in the treatment success group were. The total antimicrobial therapy duration and AUC_ss,0–24h_ of PMB were significantly greater (*p* < 0.05) in the treatment success group than in the treatment failure group; however, prolonging PMB exposure did not seem to guarantee treatment success.

Considering the limited sample size (a stringent threshold *p* < 0.05 would risk insufficient statistical power to detect potentially meaningful associations given the small sample) and the fundamental purpose of our study (in early exploratory investigations, the priority is to identify candidate variables for further scrutiny rather than definitively confirm effects; a slightly lenient threshold minimizes the risk of overlooking influential factors), individual variables with a *p*-value less than 0.1 in Table 1 were included in the multivariate regression. Multivariate logistic regression analysis revealed no significant correlations between PMB efficacy and any risk factors.

### 2.2. PK Results

**Figure 2:**
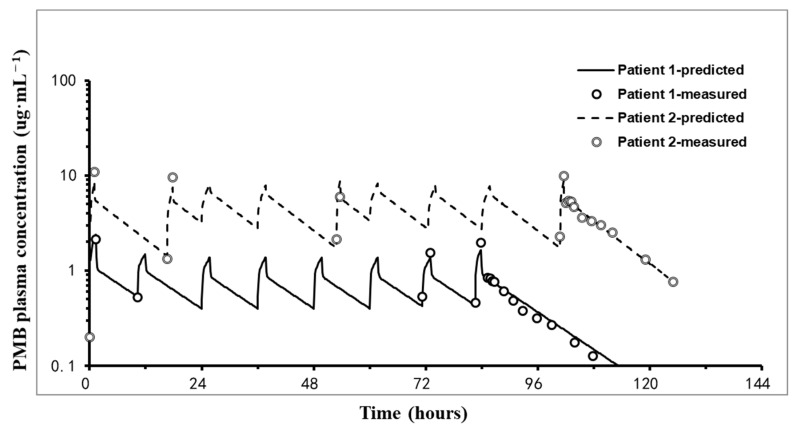
Representative fits of the pharmacokinetic model (two-compartment model) to the plasma polymyxin B concentration–time data of individual patients. The lines represent the model-predicted concentration–time curve. The circles represent the measured values.

The corresponding PK parameters of PMB derived from noncompartmental analyses after the last dose are shown in Table 2. The mean values of C_max_, t_1/2_, Vd, CL, and AUC_ss,0–12h_ were 8.3 µg/mL, 19.56 h, 30.44 L, 1.55 L/h, and 110.08 h·µg/mL, respectively. The mean plasma protein-binding rate of PMB in the serum was 85.53% at 37°C. The mean PMB concentrations after the first loading dose and at the steady state were 5.51 ug·mL^−1^ and 5.11 ug·mL^−1^, respectively. The coefficient of variation (CV) was controlled below 30%. Two typical fitted patient pharmacokinetic profiles are shown in Figure 2.

### 2.3. Pulmonary Concentrations

BAL samples were collected from 15 patients, among whom pulmonary concentrations of PMB were ultimately measured. The main reasons for excluding samples were the presence of blood (two samples) and excessive dilution (one sample below the detection limit). The mean recovery rate of BAL fluid was 25.3%. The dilution of urea in BAL fluid was 30.99 (30.47, 33.95). The median and IQR ELF concentrations were 0.90 (0.89–1.04) mg/L. The median and IQR plasma C_max_ were 7.46 [6.16, 11.34] mg/L. The median and IQR ELF/plasma ratios (%) were 15.69 [16.86, 18.15]. Three patients experienced treatment failure, while the other nine had treatment success. There was a significant difference between the treatment success and treatment failure groups in terms of the ELF/plasma ratio (0.17 [0.15, 0.20] vs. 0.088 [0.087, 0.091], *p* = 0.011) and C_max_ (5.94 [4.2, 8.77] mg/L vs. 11.12 [10.28, 11.61] mg/L, *p* = 0.016). There was no significant difference in the PMB concentration in the ELF between the two groups (0.84 [0.73, 1.08] mg/L vs. 0.98 [0.93, 1.00] mg/L, *p* = 0.76).

### 2.4. PK/PD Targets Attainment and Clinical Efficacy

As shown in Table 3, considering a target AUC_ss,0–24h_ of 50–100 h, 48.15% (13/27) of patients reached the PK/PD targets, among whom 69.23% (9/13) of patients achieved treatment success. Considering a target C_ss,avg_ of 2–4 mg/L, 33.33% (9/27) of patients reached the PK/PD targets, 77.78% (7/9) of whom achieved the desired treatment success. In addition, overexposure occurred much more frequently than underexposure (3.70% vs. 48.15% considering a target AUC_ss,0–24h_, 3.70% vs. 62.96% considering a target C_ss,avg_) did.

**Figure 3:**
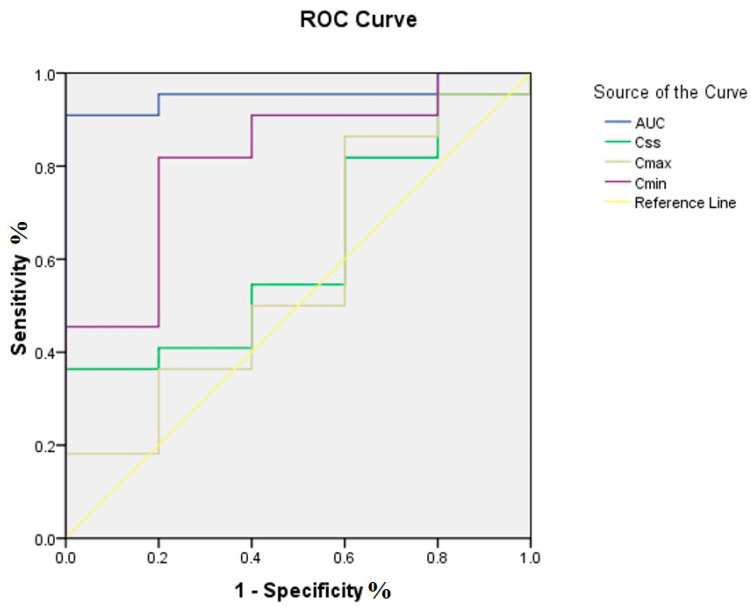
Receiver operating characteristic (ROC) curves of different PK indices for the prediction of clinical efficacy. AUC_ss,0–24h_ was superior to C_ss_, C_min_, and C_max_ for the prediction of clinical efficacy.

The analysis of the ROC curves suggested that AUC_ss,0–24h_ (AUCROC = 0.955) was superior to C_ss_ (AUCROC = 0.618), C_min_ (AUCROC = 0.818), and C_max_ (AUCROC = 0.573) for the prediction of clinical efficacy. When the Youden index was the greatest (0.909), the corresponding optimal cutoff point of AUC_ss,0–24h_ was 77.27. The predictive sensitivity and specificity of these values were 90.9% and 100%, respectively (Figure 3).

### 2.5. Correlations Between Patients’ Candidate Biomarkers and PK Parameters

As shown in Table 4, tumour necrosis factor-α (TNF-α) was negatively correlated with the ELF/plasma ratio (*p* = 0.014) and positively correlated with Vd (*p* = 0.034). The amount of produced IL-10 varies widely between individuals. For a better comparison of the pro- versus anti-inflammatory balance, the ratio of interleukin-6 (IL-6) to interleukin-10 (IL-10) has been suggested [43,44]. A higher ratio of IL-6 to IL-10 indicates a trend toward a proinflammatory milieu. The IL-6/IL-10 ratio was negatively correlated with the trough steady-state concentration (*p* = 0.042) and t_1/2_ (*p* = 0.034).

## 3. Discussion

Overall, our findings indicate that achieving optimal PK/PD targets correlates is associated with improved outcomes, thereby supporting the rationale for biomarker-driven therapeutic strategies. This study evaluated the PK parameters and PD responses in critically ill patients receiving intravenous PMB through a simplified fixed-dose regimen, as outlined in the drug instructions, specifically, a 100 mg loading dose followed by 50 mg maintenance doses every 12 h. The regimen was notably lower than the total body weight (TBW)-based dose recommended by ICGOUP, which suggests a loading dose of 2–2.5 mg/kg and a maintenance dose of 1.25–1.5 mg/kg. PMB is a concentration-dependent antibiotic. For a concentration-dependent antibiotic, the best PK/PD target is used to be fAUC0–24/MIC. However, AUC_ss,0–24h_ and Css,avg are recommended as PK/PD therapeutic targets for PMB by ICGOUP because concentrations higher than this were shown to increase both the incidence and severity of AKI. This is acceptable from a toxicity standpoint. The PK profiles of PMB in our study exhibited significant alterations, including a prolonged half-life (t_1/2_) and higher maximum concentration (C_max_) and area under the concentration–time curve over 24 h at steady state (AUC_ss,0–24h_) compared to previously reported data, as detailed in Table A1 [45,46,47,48,49,50,51,52,53,54,55,56,57,58,59,60]. These discrepancies may be attributed to variations in patient demographics, ethnic backgrounds, therapeutic regimens, potential drug-drug interactions, analytical methodologies, sampling protocols (whether sparse or intensive), software used for PK modeling, and the structural assumptions of the PK models employed across different studies [7]. Notably, factors such as tumor necrosis factor-alpha (TNF-α) concentrations and the interleukin-6 to interleukin-10 (IL-6/IL-10) ratio were found to influence PK parameters, a finding that has not been previously documented in the literature.

Despite the lower drug exposure observed in the epithelial lining fluid (ELF), the simplified fixed-dose regimen demonstrated efficacy in critically ill patients suffering from extensively drug-resistant Gram-negative (XDR-GN) bacterial lung infections. This suggests that the regimen, while suboptimal in terms of ELF penetration, may still provide sufficient systematic exposure to achieve therapeutic outcomes in this patient population.

### 3.1. PK–PD Characteristics

Both one- and two-compartment methods have been described in critically ill patients [45,46,47,48,49,50,51,52,53,54,55,56,57,58,59,60]. The one-compartment PK model from other studies might be explained by the lower Vd. Penetration into the peripheral compartment might rarely be observed within a short period when the blood sample is collected after one dose. In our study, an intensive sampling method was adopted, and the PMB PK parameters were best described by a two-compartment model.

PMB plasma concentrations, both after the loading dose and at the steady state, were greater in our study [49,53,56,57,58,59,60] than in other studies, although the administration dosage in our study was lower [47,48,49,53,56,57,58,59,60]. These differences may be attributed to the relatively short infusion time in our study (0.5–1 h) compared with that in other studies (>1–2 h) [45,46,47,48,49,50,56,59,60]. In most studies, steady-state trough serum concentrations were sampled after patients were treated with PMB for 48 h [51,52,53,54,59] or 72 h [50,57,60]. In our study, considering the special condition of critically ill patients, the steady-state trough serum concentration was sampled before the initiation of the seventh dose (on day 4 of treatment). The analytical methods used might also contribute to these differences. Most of the studies have quantitatively calculated the PMB concentration as the sum of PB1 and PB2 [45,48,49,50,54,55,57,59] via LC–MS/MS, whereas others have calculated the PMB concentration via ELISA [53]. In our study, the sum of PB1, PB2, PB3, and I-PB1 was used.

The Vd in our study was consistent with what has been reported for other populations and might result from timely amelioration of low blood albumin levels, optimization of fluid management, and a slight change in plasma protein binding (PPB) [61]. We also discovered a positive relationship between the TNF-α level and Vd. The release of inflammatory mediators increased vascular permeability, promoted fluid entry into the third space, and increased the PMB Vd.

The t_1/2_ values in our study were all higher than those reported in other studies [47,58]. Our studies revealed that the ratio of IL-6/IL-10 was negatively correlated with the trough steady-state concentration and t_1/2_, indicating that disruption of the proinflammatory versus anti-inflammatory balance accelerated the elimination of PMB and thus decreased the trough steady-state concentration. In this study, the t_1/2_ was calculated with blood samples collected after the last administration, during which 81.48% (22 out of 27) of the patients experienced treatment success and a restoration of inflammatory homeostasis. These findings may have been one of the reasons leading to the greater t_1/2_ and AUC_ss,0–24h_ in our study.

The estimated CL (1.55 L/h) was lower than the values reported in American critically ill patients with Gram-negative infections (approximately 2.5 L/h) [45,46,52] and in Chinese critically ill patients receiving continuous renal replacement therapy (CRRT) (1.95 L/h) [51] but equivalent to the values reported in other Chinese studies [50,51,53,56,57,59,60].

In previous studies, many factors, such as age, sex, TBW, ideal body weight, plasma ALB concentration, creatinine clearance, and APACHE II score, were investigated as covariates during the modelling process [53,54,56]. Our research revealed that factors such as the TNF-α concentration and the IL-6/IL-10 ratio also affect PK parameters, so they should be considered important covariates in future studies.

In addition to acute illness-mediated changes in PK, interethnic differences may also contribute to differences in PK parameters. The influence of ethnicity on drug PK has been reported for several medicines, such as cyclosporine, methadone, and efavirenz [62,63,64,65,66,67,68,69]. Interethnic PK differences may stem from differences in body composition [70,71,72], protein-binding capacity [73,74], or disposition capacity due to genetic polymorphisms [75,76,77,78,79]. For example, in non-ICU studies, the reported mean Vd values of linezolid in Asian and non-Asian patients were 0.59 L/kg and 0.58 L/kg, respectively [80,81]. However, ICU studies have shown a marked difference in the mean Vd of linezolid in the central compartment between Asian and non-Asian patients (0.34 L/kg vs. 0.19 L/kg) [82,83].

Interethnic differences may also exist in PMB pharmacokinetics. According to noncritically ill studies, non-Asian patients had a higher CL (2.63 L/h in Miglis’s study, 2.37 L/h in Kubin’s study, and 2.5 L/h in Manchandani’s study) [45,46,47] than Asian patients did (1.79 L/h in Wang’s study and 1.59 in Yu’s study) [50,51]. According to critically ill MDR studies, the CL rates reported in non-Asian patients and Asian patients were 1.76 L/h (in Surovoy’s study) [53] and 1.55 L/h (in our study), respectively. In studies of noncritically ill patients, the Vd reported in non-Asian patients (33.77 in Miglis’s study, 34.4 L in Kubin’s study, and 34.3 L in Manchandani’s study) [45,46,47] was greater than that reported in Asian patients (20.5 in Yu’s study) [51]. In studies of critically ill patients, the Vd reported in non-Asian patients and Asian patients was 30.4 L (in Surovoy’s study) [53] and 30.44 L (in our study), respectively.

Finally, we cannot rule out the possibility of the potential impacts of other combined drugs on PMB PK and the heterogeneity among the patients in our study. To date, little evidence supports the conclusion that the combined use of other drugs affects PMB PK. Studies have shown that urinary recovery from PMB is low (<5%) [61]. Although the mechanism of the nonrenal clearance of PMB has not been fully elucidated, biliary excretion has been suggested, as all four components of PB have been detected in bile [84]. Further studies examining the nonrenal routes of PMB elimination are warranted, which may further promote drug interaction research.

### 3.2. Biomarkers Predicting Tissue Pharmacokinetics

The intricate pathophysiology of critically ill patients, coupled with the limited efficacy of current targeted therapies, underscores the need for novel biomarkers capable of predicting target site drug concentrations. Such biomarkers could guide drug dosing and provide real-time feedback on therapeutic efficacy in this vulnerable population. Our study aimed to elucidate the mechanisms underlying PK processes, with a particular focus on how pathophysiological changes influence PK alterations and how biomarkers might serve as predictive tools. In critically ill patients with infections, the immune system often responds to pathogens by releasing proinflammatory factors, including IL-1β, IL-6, IL-18, interferon, and TNF-α [85]. Uncontrolled inflammation can exacerbate tissue edema, capillary leakage, organ failure, and shock, all of which may significantly alter drug distribution and PK profiles at target sites. Our findings reveal that TNF-α levels are negatively correlated with the ELF/plasma ratio and positively correlated with the Vd of PMB. Additionally, the production of the anti-inflammatory cytokine IL-10 varies considerably among individuals. To better assess the balance between pro- and anti-inflammatory responses, the ratio of IL-6 to IL-10 has been proposed as a useful metric [43,44]. Notably, the IL-6/IL-10 ratio demonstrated a statistically significant positive correlation with the C_ss,trough_ and a negative correlation with the t_1/2_ of PMB. Further high-quality research is needed to clarify the potential role of cytokine profiles as predictive biomarkers, which may be useful for optimizing drug dosing and therapeutic outcomes in critically ill patients.

Inflammatory factors have emerged as valuable biomarkers for the early diagnosis and prognosis of sepsis [86,87,88]. Under normal conditions, inflammatory responses lead to fluid extravasation and an increase in the Vd for hydrophilic antibiotics [89]. However, our data reveal a negative correlation between TNF-α levels and both the ELF/plasma ratio and the Vd of PMB. We hypothesize that excessive inflammation may disrupt microcirculation, impairing its structural and functional integrity. This disruption compromises the ability of microcirculation to meet the metabolic demands of tissues and organs, thereby hindering the exchange of materials between blood and tissues.

Immunological biomarkers are increasingly recognized as indicators of effective antimicrobial therapy [90], as they may correlate with drug concentrations at target sites. While some studies have suggested the potential of immunological biomarkers to guide antimicrobial treatment, the specific relationships remain poorly understood [91,92]. In our study, we observed a negative correlation between TNF-α levels and the ELF/plasma ratio, suggesting that overactivation of TNF-α-mediated downstream responses may not enhance physiological barriers or improve tissue penetration. These findings prompt us to pay attention to the complex interplay between inflammation, microcirculation, and drug distribution. Further research is needed to clarify these mechanisms and clarify how to optimize therapeutic strategies through intervention of these factors in critically ill patients.

### 3.3. PMB Penetration into the Lung

Although very high concentrations of the formed CMS or PMB in the ELF have been reported after aerosolized PM delivery, with or without intravenous administration in critically ill patients [39,93,94], less is known about the pulmonary penetration rate of PMB after intravenous administration. Our study revealed that the median (IQR) ELF concentration and ELF/plasma ratio (%) of PMB were 0.90 [0.89–1.04] mg/L and 15.69 [16.86, 18.15], respectively. The ELF/plasma ratio differed between the treatment success group and the treatment failure group (0.17 [0.15, 0.20] vs. 0.088 [0.087, 0.091], *p* = 0.011), indicating marked differences between patients. Similar findings have been reported in other studies. The concentrations of linezolid in ELF and macrophage ranged from 13.1 to 52.4 mg/mL and 0.5 to 23.7 mg/mL, respectively [95]. Many factors influence antimicrobial agent penetration into pulmonary compartments [96,97,98,99,100,101,102], such as the blood–bronchus barrier, protein-binding rate, lipophilicity, pH, and inflammation at the site of infection. [94,96]. For example, Lamer et al. [103] reported that patients with lung inflammation (ELF albumin concentration ≥ 3.4 mg/mL vs. <3.4 mg/mL) had significantly greater vancomycin ELF/plasma values than patients without lung inflammation did (0.246 [0.192–0.426] vs. 0.14 [0.023–0.285]). Drug transporter systems may also be involved [104,105,106,107]. Technologies related to sample collection and quantification also influence lung penetration [106,107,108]. Since the drug concentrations in plasma and ELF change with sampling time and place, it is recommended to calculate penetration ratios with the ELF AUC and plasma AUC.

The literature consistently reports very low or undetectable levels of PMB in the ELF [28,29]. In our study, we similarly observed poor ELF penetration, which may be attributed to the compound’s polar nature, partial ionization at physiological pH, and high molecular weight. Additionally, PMB exhibits a high plasma protein-binding capacity, ranging from 80% to 90%, as confirmed by our findings and those of others. These factors collectively limit its distribution into the ELF. Some of the observed discrepancies in ELF concentrations may also stem from differences in treatment regimens. For instance, PMB accumulation in the ELF after a single dose is likely significantly lower than after multiple doses. In our study, ELF concentrations were measured following multiple doses, which may partially explain the variability compared to single-dose studies. Furthermore, differences in analytical methodologies could contribute to these variations. It is crucial that the analytical methods employed are both highly sensitive and specific to ensure accurate quantification of PMB levels in complex biological matrices like ELF. These considerations highlight the need for standardized approaches in future research to better understand and optimize PMB distribution in critically ill patients.

Notably, although the reported level of PMB was low in ELF, the response rate to PMB in treating lung infection was 81.48% in our study. In the case of lung infection, PMB from alveolar macrophages and neutrophils was also imported and not examined in our study or other studies. For instance, the azalide antibiotic azithromycin exhibits unique pharmacokinetic properties, characterized by exceptionally high concentrations in alveolar macrophages but very low plasma levels [108,109]. PMs, a group of alkaline polypeptides, may similarly accumulate in phagocytic cells. Basic compounds like PMs can become protonated and subsequently trapped within lysosomes of phagocytes [110]. This raises the possibility that macrophages loaded with antimicrobial agents could serve as delivery systems, transporting these agents to localized sites of infection within the lung [111]. Further research to quantify PMB concentrations in alveolar macrophages and neutrophils could provide valuable insights into its mechanism of action in treating lung infections.

Additionally, certain antimicrobial agents may exert anti-inflammatory effects that are independent of their bacterial clearance properties. For example, erythromycin has demonstrated clinical benefits in patients with diffuse panbronchiolitis, irrespective of *Pseudomonas aeruginosa* infection. Erythromycin therapy has been associated with improved lung function, enhanced gas exchange, and reduced neutrophil counts in BAL fluid [112]. Even at low doses, erythromycin—and potentially other macrolides—may inhibit cytokine production, such as IL-8, by neutrophils. This suggests a broader immunomodulatory role for these agents beyond their antimicrobial activity.

Moreover, there is a mechanistic rationale for combining PMs with other antimicrobial agents that exhibit synergistic effects. PMs, as membrane permeabilizers, may enhance the intracellular uptake of companion antibacterial agents targeting intracellular pathogens [113,114,115,116,117]. In our previous study, we found that among PMB-based combinations, the pairing of PMB with sulbactam (SB) demonstrated superior therapeutic efficacy at low PMB doses in critically ill patients with carbapenem-resistant *Acinetobacter baumannii* nosocomial pneumonia [113]. These findings highlight the potential of combination therapies to optimize treatment outcomes.

Finally, exploring alternative delivery methods for PMB to improve its pulmonary penetration could further enhance its efficacy in treating lung infections. Such approaches may include novel formulations or targeted delivery systems designed to maximize drug concentrations at the site of infection while minimizing systematic exposure.

### 3.4. PMB Safety

Nephrotoxicity is a primary side effect of PMB. However, in this study, the research subjects were critically ill patients, among whom it was difficult to make causal decisions regarding adverse reactions. Thus, we recorded the number of patients (only four patients) who suffered from nephrotoxicity when PMB medication was administered. Determining the specific causes based on medication information and pathophysiological state is a complex process. We also explored the effects of the PMB dosage and exposure (plasma concentration) on nephrotoxicity. Only one of the four patients overexposed to PMB achieved treatment success. The small sample size is difficult to analyze with statistical methods.

### 3.5. Limitations

This study has several limitations. First, we adopted a simplified dose of PMB, and we do not know whether lower or higher doses would result in better PK/PD. Second, the PMB concentrations in alveolar macrophages and neutrophils were not determined, which may underestimate the target site concentrations and clinical efficacy of the treatment. Third, BAL fluid from the most severely ill patients was not sampled, which may also introduce bias to explain the results. Fourth, the lack of pharmacogenomic assessment is another limitation. Finally, the relatively small sample size was limited for PK/PD evaluation, especially for optimizing the PMB PK/PD targets. With our dataset of 22 (treatment success) and our dataset of 5 (treatment failure), the sample size difference is still a bit large. This can have an impact on the efficiency of the test, especially since the results of the small sample group may be unstable. Fortunately, there were no extreme values in the distribution of small sample size groups, and all the samples in the treatment failure group had certain representativeness. To some extent, our results could be used as reference for clinical practice.

## 4. Materials and Methods

### 4.1. Patients and Ethics

A prospective cohort study was performed in adult (age ≥ 18 years) patients, whose etiological cultures suggest XDR-GN infection (drug sensitivity results suggest it was sensitive to PMB), treated with intravenous PMB (sulfate, polymyxin B for injection, SPH No. 1 Biochemical & Pharmaceutical Co., Ltd., Shanghai, China) for more than 3 days from September 2022 to December 2024 at Peking Union Medical College Hospital, Beijing, China. The decision to administer PMB was made by the attending physicians. Patients with conditions that could alter the PK of PMB, who had rapidly declining or fluctuating renal function (serum creatinine ± 50% since the start of therapy, who could affect the removal of drugs from the body), who were pregnant (hormone levels, increased blood volume, and other factors affecting drug processes), who had severe burns/head/spinal cord injury (high metabolic status, fluid redistribution, reduced plasma protein levels, and other factors affecting drug processes), who had cystic fibrosis (decreased distribution of PMB in lungs), or who were severely under- or overweight (±40% of ideal body weight, changes of fat volume, and body surface area affecting drug processes) were excluded. The study was approved by the Ethics Committees of Peking Union Medical College Hospital and the Declaration of Helsinki [118]. Written informed consent was obtained from all patients or their legal representatives.

### 4.2. Data Collection

Patient characteristics and laboratory data, including age, sex, weight, medical history, Acute Physiology And Chronic Health Evaluation (APACHE) II score, Sequential Organ Failure Assessment (SOFA) score, estimated creatinine clearance, arterial blood gas analysis, life support interventions, infection site, and type of pathogen, were recorded before PMB administration. The anti-infection therapeutic regimen, course, and incidence of PMB-related adverse reactions were recorded during the study. The creatinine clearance rate (CCr) was calculated with the formula: Ccr = (140 − age) × weight (kg)/[72 × serum creatinine (mg/dL)] for male and Ccr = (140 − age) × weight(kg)/[85 × serum creatinine (mg/dL)] for female. Nephrotoxicity was defined as an increase in serum creatinine of ≥0.3 mg/dL within 48 h or an increase in serum creatinine to ≥1.5 times higher than baseline [119].

Inflammatory cytokine levels were measured by the clinical laboratory with ELISA before and 3 days after PMB administration for analysis, and these data were also collected from the hospital’s information system. All the above data were used to investigate the factors that affect the plasma concentrations of PMB and its PK parameters.

### 4.3. PMB Administration and Sample Collection

The patients received a 100 mg loading dose followed by a maintenance dose of 50 mg every 12 h. PMB was administered by short-term infusions (60 min). Blood samples were collected from each patient in three stages.

Phase of first dose: Blood samples were collected at defined time points to capture PK data.

Phase of steady state: Although a steady state of PMB concentration can be achieved after 48 h of treatment, considering the special condition of critically ill patients, blood samples were collected immediately before the initiation of the seventh dose (trough) and immediately at the end of the seventh dose (peak).

During the last dose phase, blood samples were collected immediately before the initiation of the last dose, immediately at the end of the last dose, and randomly (at least eight blood samples for each person) at 1.5, 2, 2.5, 3, 5, 7, 9, 12, 15, 19, 24, 48, and 72 h after completing the infusion.

BAL sample collection was possible for 15 of the patients. If the patients’ respiratory status permitted, one BAL was performed immediately after the seventh dose (on day 4 of treatment). Four 20 mL aliquots of sterile 0.9% normal saline solution were instilled, immediately aspirated, and placed on ice. The aspirate from the first 20 mL instillation was collected and discarded. The aspirates recovered from the second, third, and fourth instillations were pooled.

Blood and BAL samples were centrifuged for 10 min (3000 rpm) and immediately stored at −80 °C until analysis.

### 4.4. Quantification of PMB Concentrations in Plasma and BAL Fluid

PMB concentrations in the samples were quantified by monitoring the total concentrations of polymyxin B1 (PB1), polymyxin B2 (PB2), polymyxin B3 (PB3), and isoleucine polymyxin B1 (I-PB1) via a validated ultraperformance liquid chromatography–tandem mass spectrometry (UPLC-MS/MS) assay [58]. The standard substances, including PB1 (CAS P037), PB2 (CAS P037), PB3 (CAS P040), and I-PB1 (CAS P038), were purchased by TOKU-E Company (>99% pure, Bellingham, WA, USA). ACQUITY UPLC system, Quattro PremierXE triple quadrupole mass spectrometer, electrospray isonizaiton source (ESI), and Masslynx 4.1 data processing software were used in this study (Waters Company, Milford, MA, USA). An Acquity UPLC BEH C18 column (50 mm × 2.1 mm internal diameter, 1.8 μm) purchased from Waters (Milford, MA, USA) was used. The mobile phase comprised a 0.2% formic acid aqueous solution and a 0.2% formic acid acetonitrile solution, and the flow rate was 0.3 mL/min. Mass spectrometric detection was performed in positive electrospray ionization (ESI+) mode with multiple reaction monitoring (MRM). The optimized transitions were set as follows: *m*/*z* 602.7→101.0 for PB1 and I-PB1, *m*/*z* 595.7→100.9 for PB2 and PB3, and *m*/*z* 595.7→100.9 for the internal standard norvancomycin. Plasma and BAL supernatant was aliquoted into microcentrifuge tubes and sequentially treated with 20 μL norvancomycin or 200 μL 5% trichloroacetic acid. 1 μL plasma and 2 μL BAL of supernatant were collected separately for the detection of PMB concentrations.

Analysis of independently prepared quality control samples indicated good reproducibility (coefficients of variation ≤ 8.39%) and accuracy (measured concentrations ≤ 10.0% from target concentrations). The standard curve was linear over the range of 0.5 to 10,000 ng/mL, with precision and accuracy of less than 15%. The regression coefficient was 0.9985. The relative standard deviation values for the intra- and inter-day precision at low, middle, and high levels were all less than 6%, and the recovery rate was 83.1–85.4%. The matrix effect and the stability of whole blood, plasma, and freeze-thaw cycle met the analytical requirements, and the methodology was shown to be stable and reliable.

### 4.5. Quantification of PMB Concentrations in ELF

The ELF recovery rate was calculated via the urea dilution method, as reported in previous studies [120,121,122]. The concentration of urea was analysed with a commercial kit purchased from Beijing Solarbio Science & Technology Co., Ltd., Beijing, China (BC1535). The concentration of PMB in the ELF (ELF[PMB]) was measured by using urea as an endogenous marker of ELF dilution via the following equation: ELF[PMB] = BAL[PMB] × (blood urea/BAL[Urea]), where BAL[PMB] is the concentration of PMB in BAL and BAL[Urea] is the concentration of urea in BAL.

### 4.6. Binding of PMB in Plasma

The plasma protein binding of PMB was determined by rapid equilibrium dialysis device purchased from Thermo Scientific™ (Pierce RED, CAS90006, Rockford, IL, USA) [123]. For each patient, three plasma samples (totaling approximately 1 ml) were pooled (pH adjusted to 7.4), and the test was performed in triplicate. For each, 300 μL was dialyzed against an equal volume of isotonic phosphate-buffered saline (pH 7.4) at 37 °C for 4 h. PMB concentrations were determined, and the extent of protein binding was calculated as 1 minus the ratio of the PMB concentration in the buffer to that in the plasma and is expressed as a percentage.

### 4.7. PK Analysis

The plasma concentration–time values for PMB were analysed via Phoenix WinNonlin 8.1. The data were explored via noncompartmental and two-compartment PK models. The principles for final model selection were a low Akaike information criterion (AIC), visual evaluation of individual plasma concentration–time profiles, and precision of parameter estimations on the basis of one sample t-test or the correlation coefficient. The following PK parameters were generated via noncompartmental and two-compartment analysis: C_max_, CL, Vd, t_1/2_, k, and AUC was determined via the linear trapezoidal rule. The PMB C_ss,avg_ was calculated from the C_max_ and C_min_ that were directly obtained from the observed peak and trough concentrations in the stable state. The simulated concentrations were obtained via compartment PK models. Factors that affect PMB PK parameters were also evaluated.

### 4.8. PD Study

The primary PD response was evaluated by PK/PD indices. An AUC_ss,0–24h_ of 50–100 mg·hour/L or a C_ss,avg_ of 2–4 mg/L was used as the PK/PD targets for PMB. The secondary PD response was evaluated by clinical parameters. An achievement of the treatment success at the end of treatment was defined by either (1) physicians’ judgment of improvement in signs and symptoms (reasonable compromise on the basis of survival, stable, or improved haemodynamics and oxygenation index and improved chemical and physiological indices of infection) or (2) microbiological elimination. Failure in patients who did not meet the above criteria was defined as treatment failure. The percentage of patients who achieved the PK/PD targets or treatment success was calculated. Variables potentially related to clinical efficacy were assessed. Receiver operating characteristic (ROC) curves were drawn to investigate and compare different indices as predictors of clinical efficacy. The optimal cutoff point for the PK/PD indices was calculated with the Youden index.

### 4.9. Statistical Analysis

Normally distributed data are presented as the mean ± standard deviation (SD) and were compared via Student’s t-test. Nonnormally distributed data are reported as the median and interquartile range (IQR) and were analyzed via the Mann–Whitney U test. Logistic regression analysis was conducted to evaluate the associations between ELT efficacy or adverse drug reactions (ADRs) and ELT concentrations by adjusting for potential confounding factors. Spearman’s rho correlation coefficient and Pearson’s bivariate correlation coefficient were calculated to identify factors contributing to the PD data.

Our study is a prospective study of a relatively rare clinical cohort. The number of eligible patients who met the inclusion criteria during the study period was limited, leading to a final sample size of 27 patients. Given this constraint, we conducted a sample size estimation to assess the statistical power of our study. For continuous variables, assuming standard deviations of 5, 10, and 15, our sample size provides 80% power to detect group differences of 7.2, 14.4, and 21.7, respectively. For categorical variables, assuming an exposure prevalence of 20%, this sample size provides 80% power to detect a relative risk (RR) increase of three-fold or greater (RR = 4).

All the statistical data were analyzed via SPSS (IBM Corp. Released 2019. IBM SPSS Statistics for Windows, Version 26.0. Armonk, NY, USA: IBM Corp.) [124]. A *p*-value (95% confidence interval [CI]) less than 0.05 was considered significant.

## 5. Conclusions

Clarifying the PK/PD relationship in critically ill patients is a necessary challenge. This is the first study to investigate the steady-state percentage penetration of a simplified fixed dose of 50 mg of PMB systematically administered every 12 h after a 100 mg loading dose during the treatment of severe infections in critically ill patients. PMB has definite therapeutic efficacy on nosocomial pneumonia caused by XDR-GN bacteria, although the degree of tissue exposure to PMB is relatively low. Among the other PK/PD indices we examined, AUC_ss,0–24h_ was the most reliable, with a target of 77.27. The AUC_ss,0–24h_ and C_ss_ values exceeded their recommended PK/PD targets and showed good therapeutic efficacy and safety. Our research is also the first to demonstrate the specific contribution of inflammatory factor levels (such as the TNF-α concentration and the IL-6/IL-10 ratio) to the wide interindividual variability in the PMB PK/PD profile among severely infected patients.

## Figures and Tables

**Table 1:** Baseline characteristics of the patients who received PMB therapy at PUMCH.

Demographics	Total (*n* = 27)	Treatment Success (*n* = 22)	Treatment Failure (*n* = 5)	*p*
Age (years) [IQR] ^a^	68.0 [63.5, 74.5]	67.5 [56.8, 72.0]	75.0 [74.0, 75.0]	0.218
Male sex, *n* (%) ^b^				0.618
Male	19 (70.4)	15 (68.2)	4 (80.0)	
Female	8 (29.6)	7 (31.8)	1 (20.0)	
Weight (kg) [IQR] ^a^	67.5 [60.0, 76.5]	66.3 [60.0, 78.5]	68.0 [56.0, 70.0]	0.430
BMI [IQR] ^a^	24.2 [22.2, 25.7]	24.3 [22.6, 25.9]	22.7 [21.9, 24.0]	0.274
Baseline CCr (mL/min) [IQR ] ^a^	72.5 [34.4–125.6]	72.8 [34.0–125.4]	77.8 [45.5–136.7]	0.350
Cardiovascular diseases, *n* (%) ^b^	14 (51.9)	11 (50.0)	3 (60.0)	0.574
Diabetes, *n* (%) ^b^	6 (22.2)	4 (18.2)	2 (40.0)	0.320
Renal dysfunction, *n* (%) ^b^	12 (44.4)	9 (40.9)	3 (60.0)	0.458
Hepatic dysfunction, *n* (%) ^b^	5 (18.5)	4 (18.2)	1 (20.0)	0.510
APACHE II score [IQR] ^a^	22.0 [17.0, 24.0]	19.5 [16.0, 22.0]	22.0 [20.0, 24.0]	0.049
SOFA score [IQR] ^a^	12.0 [9.0, 14.0]	11.0 [9.0, 13.5]	12.5 [9.5, 15.5]	0.672
Mechanical ventilation, *n* (%) ^b^	25 (92.6)	20 (90.9)	5 (100.0)	0.502
CRRT, *n* (%) ^b^	9 (33.3)	8 (36.4)	1 (20.0)	0.460
ECMO, *n* (%) ^b^	1 (3.7)	1 (4.6)	0 (0.0)	0.643
Septic shock, *n* (%) ^b^	13 (48.2)	10 (45.5)	3 (60.0)	0.574
MODS, *n* (%) ^b^	6 (22.2)	4 (18.2)	2 (40.0)	0.340
LOS (days) [IQR] ^a^	25 [16.0, 50.5]	22.0 [16.3, 43.3]	25.5 [16.0, 54.0]	0.155
ICU LOS (days) [IQR] ^a^	22 [12.0, 30.0]	21 [9.3, 26.0]	22 [16.0, 34.0]	0.113
Antimicrobial therapy duration (days) [IQR] ^a^	20 [12.0, 27.0]	21 [9.3, 27.5]	16 [16.0, 22.0]	0.363
PMB therapy duration (days) [IQR] ^a^	9 [5.0, 14.0]	7 [4.3, 13.0]	13 [13.0, 15.0]	0.002
PMB PK/PD indices [IQR] ^a^				
AUC_ss,0–24h_	100.8 [76.9, 137.3]	111.7 [85.5, 146.9]	64.7 [58.9, 75.1]	0.002
C_ss,avg_	5.2 [3.8, 5.9]	5.0 [3.7, 5.5]	6.6 [5.9, 7.7]	0.903
C_ss,max_	8.7 [6.6, 10.1]	8.7 [6.1, 9.7]	11.1 [9.9, 11.4]	0.647
C_ss,min_	2.2 [1.6, 3.1]	2.1 [1.1, 2.9]	3.00 [2.9, 3.1]	0.529
Anti-GNB regimens, *n* (%) ^b^				
Monotherapy	4 (14.8)	4 (18.2)	0 (0.0)	1.000
Two-drug combination				
PMB + Carbapenase	9 (33.3)	7 (31.8)	2 (40.0)	1.000
PMB + β-lactam or β-lactam/β-lactamase Inhibitors	3 (11.1)	3 (13.6)	0 (0.0)	1.000
PMB + Tetracycline	1 (3.7)	1 (4.6)	0 (0.0)	1.000
PMB + Fluoroquinolone	3 (11.1)	3 (13.6)	0 (0.0)	1.000
Three or more drug combination	7 (25.9)	4 (18.2)	3 (60.0)	0.101
Isolated GNM, *n* (%) ^b^				
*Acinetobacter baumannii*	14 (51.9)	12 (54.6)	2 (40.0)	0.648
*Pseudomonas aeruginosa*	5 (18.5)	3 (13.6)	2 (40.0)	1.000
*Klebsiella* spp.	9 (33.3)	8 (36.4)	1 (20.0)	0.636
*E. coli*	2 (7.4)	2 (9.1)	0 (0.0)	1.000
*Burkholderia cepacia*	6 (22.2)	4 (18.2)	2 (40.0)	0.303
*Stenotrophomonas maltophilia*	3 (11.1)	2 (9.1)	1 (20.0)	0.474
Others	6 (22.2)	5 (22.7)	1 (20.0.)	1.000
Isolated GPM, *n* (%) ^b^	8 (29.6)	5 (22.7)	3 (60.0)	0.136
Multisites, *n* (%) ^b^				
Blood	6 (22.2)	2 (9.1)	4 (80.0)	0.004
Others	9 (33.3)	9 (40.9)	0 (0.0)	0.136
Inflammatory factors before PMB [IQR] ^a^				
TNF-α, (pg/mL)	17.6 [11.5, 22.5]	16.6 [11.1, 21.4]	17.8 [17.5, 23.5]	0.844
IL-6/IL-10	7.7 [2.71, 11.66]	7.91 [2.9, 14.7]	5.22 [2.2, 10.8]	0.424
Inflammatory factors after 3 days of PMB [IQR] ^a^				
TNF-α, (pg/mL)	20.6 [10.25, 24.80]	20.2 [10.0, 25.5]	21.6 [19.5, 22.6]	0.604
IL-6/IL-10	6.6 [2.36, 13.59]	6.14 [2.0, 13.0]	8.3 [6.1, 18.3]	0.740
Nephrotoxicity, *n* (%) ^b^	4 (14.81)	2 (9.1)	3 (20.0)	0.461

AUC_ss,0–24h_, steady-state area under the plasma concentration–time curve from time 0 to 24 h; BMI: body mass index; C_ss,avg_, average concentration at steady state; C_ss,max_, maximum concentration at steady state; C_ss,min_, minimum concentration at steady state; CCr: creatinine clearance rate; CRRT: continuous renal replacement therapy; ECMO: extracorporeal membrane oxygenation; PMB: polymyxin B; GNB: Gram-negative bacteria; GNM: Gram-negative microorganisms; GPM: Gram-positive microorganisms; ICU, intensive care unit; LOS, length of stay; MODS: multiple organ dysfunction syndrome. ^a^: Data are presented as the median (interquartile range [IQR]). ^b^: Data are presented as *n* (%). Continuous variables were analysed via Student’s *t*-test or the Mann–Whitney U test. Categorical data were analysed via the χ^2^ test or Fisher’s exact test.

**Table 2:** PK parameters in 27 enrolled patients.

Parameter	Mean	SD	CV (%)
C_ss,max_ (μg·mL^−1^)	8.3	0.7	8.4
t_1/2_ (h)	19.6	4.6	23.5
Vd (L)	30.4	6.4	21.1
CL (L·h^−1^)	1.6	0.4	25.0
C_loading dose_ (μg·mL^−1^)	5.5	3.0	5.5
C_ss,avg_ (μg·mL^−1^)	5.1	1.1	21.6
AUC_ss,0–24h_ (h·μg·mL^−1^)	110.0	8.5	15.5
PPBR (%)	85.7	5.1	6.0

AUC_ss,0–24h_: steady-state area under the plasma concentration–time curve from time 0 to 24 h; CL, drug clearance; C_loading dose_: concentration after the first loading dose; C_ss,max_, maximum concentration under steady state; C_ss avg_, average concentration under steady state; CV, coefficient of variation; PK pharmacokinetic; PPBR: plasma protein binding rate; SD, standard deviation; t_1/2_: elimination half-life; Vd, volume of distribution.

**Table 3:** Comparison of PK/PD targets attainment in different subgroups.

PK/PD Targets Attainment	Total (*n* = 27)	Treatment Success (*n* = 22)	Treatment Failure (*n* = 5)	*p*-Value
AUC_ss,0–24h_ < 50 (h·μg·mL^−1^), *n* (%)	1 (3.70)	0 (0)	1 (20.00)	0.061
AUC_ss,0–24h_ of 50–100 (h·μg·mL^−1^), *n* (%)	13 (48.15)	10 (45.45)	3 (60.00)	
AUC_ss,0–24h_ > 100 (h·μg·mL^−1^), *n* (%)	13 (48.15)	12 (54.55)	1 (60.00)	
C_ss,avg_ < 2 (μg·mL^−1^), *n* (%)	1 (3.70)	0 (0)	1 (20.00)	0.140
C_ss,av_g of 2–4 (ug·mL^−1^), *n* (%)	9 (33.33)	7 (31.82)	2 (40.00)	
C_ss,av_g > 4 (μg·mL^−1^), *n* (%)	17 (62.96)	15 (68.18)	2 (40.00)	

AUC_ss,0–24h_: area under the plasma concentration–time curve from time 0 to 24 h; C_ss,avg_: average concentration under steady-state conditions.

**Table 4:** Correlations between the patients’ candidate biomarkers and PMB PK characteristics.

Patient Factor		PMB Concentration		PK Parameters					Pulmonary Distribution
		C_pre_	C_0_	T_max_	t_1/2_	Vd	CL	AUC_ss_,_0–24h_	ELF/Plasma Ratio (%)
TNF-α	*p*-value	0.287	0.363	0.59	0.726	0.034 *	0.248	0.952	0.014 *
	Correlation	−0.146	0.182	0.109	0.071	0.42	−0.23	0.012	−0.485
IL-6/IL-10	*p*-value	0.042 *	0.114	0.881	0.034 *	0.995	0.418	0.652	0.205
	Correlation	−0.638	−0.311	−0.03	−0.62	0.023	0.162	−0.091	0.182
Albumin level	*p*-value	0.773	0.217	0.636	0.223	0.86	0.283	0.24	0.53
	Correlation	0.058	−0.246	−0.095	0.243	−0.036	−0.214	0.31	0.201

* *p*-value < 0.05 as statistically significant difference. AUC_ss,0–24h_: area under the plasma concentration–time curve from time 0 to 24 h; C_0_: blood samples were collected immediately at the end of the seventh dose; CL: plasma clearance; C_pre_: blood samples were collected immediately before the initiation of the seventh dose (on day 4 of treatment); ELF: epithelial lining fluid; t_1/2_: elimination half-life; T_max_: time to reach maximum concentration; Vd: volume of distribution.

## Data Availability

All data are contained within the article.

## Appendix A

**Table A1:** Published PMB PK/PD studies.

References	Populations	Infection Types	Regimen	Analytical Model	PK Parameters
Miglis et al., 2018 [45]	American	Acutely ill patients with suspected or documented Gram-negative infection	Loading dose = 2.79 (0.34–3.45) mg/kg/day Maintenance dose = 2.42 (0.34–3.45) mg/kg/day	Two-compartment model (PMB1 and PMB2)	V1 = 33.77 ± 15.21 L; V2 = 78.20 ± 37.46 L; CL = 2.63 ± 1.41 L/h
Kubin et al., 2018 [46]	American	Acutely ill patients with suspected or documented Gram-negative infection	Loading doses = 2.5 to 3 mg/kg Maintenance dose = 2.8 (1.5–3) mg/kg/day (infusion for at least 1 h)	One-compartment model	V1 = 34.4 L; CL = 2.37 L/h
Manchandani et al., 2018 [47]	Asian (from Thailand and Singapore), American	Patients with suspected or documented Gram-negative infection	2.1 ± 0.4 mg/kg/day and every 12 h to 24 h (infusion for over 1–3 h)	One-compartment model (PMB1, B2, B3, and isoleucine-B1)	CL = 2.5 ± 1.1 L/h; V = 34.3 ± 16.4 L; t_1/2_ = 10.1 h; AUC in patients with nephrotoxicity = 52.3 ± 14.8 mg·h/L; AUC in patients without nephrotoxicity = 45.1 ± 17.3 mg.h/L
Sandri et al., 2013 [48]	Brazilian, Australian	Critically ill patients with suspected or documented Gram-negative infection	0.45–3.38 mg/kg/day every 12 h or every 24 h (infusion for 1–4 h)	Nonlinear mixed-effects model S-ADAPT platform (PMB1 and PMB2)	AUC_ss,0–24h_ = 66.9 ± 21.6 mg.hour/L; C_ss,avg_ = 2.79 ± 0.90 mg/L; CL = 0.0276 L/h/kg; V1 = 0.0939 L/kg; V2 = 0.330 L/kg
Wang et al., 2021 [49]	Chinese	MDR Gram-negative bacterial infected patients with and without renal insufficiency	Loading dose = 100–150 mg Maintaining dose 2.0 ± 0.5 mg/kg/day (infusion for least 1 h)	Two-compartmental model (PMB1 and PMB2)	C_ss,min_ = 0.27 to 8.42 μg/mL; AUC_ss,0–24h_ = 80.14 ± 52.70 mg·h/L; C_ss,avg_ = 3.33 ± 2.20 μg/mL; Normal renal function: AUC_ss, 0–24h_ = 68.63 ± 43.43 mg·h/L; Renal insufficiency: AUC_ss,0–24h_ = 93.04 ± 59.52 mg·h/L
Wang et al., 2020 [50]	Chinese	MDR Gram-negative bacterial infected patients	Maintaining dose = 100 to 200 mg/day (infusion for 1–2 h)	One- and two-compartment models (PMB1 and PMB2)	V1 = 6.218 L; V2 = 11.922 L; CL = 1.786 L/h; AUC_ss,0–12 h_ = 43.64 ± 27.68 mg·h/L
Yu et al., 2020 [51]	Chinese	Suspected or documented Gram-negative bacterial infected patients with various renal functions	100–200 mg/day	One- and two-compartment models	CL = 1.59 ± 0.051 L/h; Vd = 20.5 ± 0.116 L
Thamlikitkul et al., 2016 [52]	Thai, American	Suspected or documented Gram-negative bacterial infected patients with and without renal insufficiency	1.5–2.5 mg/kg/day	One- and two-compartment models (PMB1, B2, B3, and isoleucine-B1)	Normal renal function: AUC = 63.5 ± 16.6 mg·h/L; CL = 2.5 ± 0.4 L/h Renal insufficiency: AUC = 56.0 ± 17.5 mg·h/L; CL = 2.0 ± 0.6 L/h
Surovoy et al., 2022 [53]	Russian	Critically ill patients with secondary bacterial infections caused by COVID-19-associated pneumonia	Loading dose = 200–300 mg Maintaining dose = 2.5–3 mg/kg/day	Noncompartmentalism model ELISA	ECMO: AUC_ss,0–12 h_ = 43.38 mg·h/L; AUC_ss,0–24h_ = 96.8 mg·h/L; C_SS,avg_ = 4.03 mg/L; C_max_ = 5.96 mg/L; C_min_ = 2.4 mg/L; T_max_ = 5 ± 24 min; Vd = 19.73 L Non-ECMO: AUC_ss,0–12 h_ = 34.7 mg·h/L; C_SS,avg_ = 2.9 mg/L; C_max_ = 5.08 mg/L; C_min_ = 2.15 mg/L; T_max_ = 5 ± 9; CL = 1.76 L/h; Vd = 30.4 L
Luo et al., 2022 [54]	Chinese	Patients with severe CRO infections	Loading dose = 1.73 (1.25–2.73) mg/kg/day; Maintain dose = 2.22 (1.52–3.00) mg/kg/day	Two-compartment model (PMB1 and PMB2)	V1 = 11.7 ± 6.1 L, V2 = 17.9 ± 17.9 L, Non-CRRT: CL = 1.5 ± 4.9 L/h, CRRT: CL = 1.95 ± 9.9 L/h
Yang et al., 2022 [55]	Chinese	Patients with CRO infections	1.5–3.0 mg/kg/day	Two-compartment model (PMB1 and PMB2)	AUC_ss,0–24h_ = 58.5 (IQR: 40.6, 77.2) mg·h/L C_0 h_ = 1.24 (IQR: 0.74, 1.93) mg/L
Yu et al., 2022 [56]	Chinese	Patients with CRKP bloodstream infections	Loading dose = 2.5 mg/kg (infusion at 3 h) Maintain dose = 1.25 mg/kg every 12 h (infusion for 2 h)	Non and two-compartment models (PMB1, B2, and isoleucine-B1)	Following the loading dose: C_min_ = 1.62 ± 0.41 mg/L; C_max_ = 5.53 ± 1.80 mg/L; C_ss,avg_ = 3.35 ± 1.06 mg/L; AUC_ss,0–24h_ = 79.6 ± 25.0 mg·h/L; CL = 1.6 L/h; V1 = 38.6 L; V2 = 7.13 L
Pi et al., 2023 [57]	Chinese	Critically ill patients with severe infection, sepsis, or septic shock	Loading dose = 100–200 mg Maintenance dose = 50–100 mg every 12 h	Two-compartment population PK model (PMB1 and PMB2)	AUC_ss,0–12 h_ = 17.73 (IQR, 4.10–76.01) mg·h/L, t_1/2_ = 5.29 (IQR: 0.72, 21.54) h, C_ss,avg_ = 1.48 (IQR: 0.34, 6.33) mg/L, C_max_ = 4.59 (IQR: 1.61, 14.53) mg/L, CL = 1.67 L/h, V1 = 7.857 L, V2 = 12.668 L
Chen et al., 2021 [58]	Chinese	Patients with severe pneumonia	50 mg every 12 h (infusion for 1 h)	Noncompartment model (PMB1, B2, B3, and isoleucine-B1)	AUC_ss,0–24h_ = 72.7 ± 28.9 mg·h/L; C_max_ = 5.5 ± 1.9 μg/mL; t_1/2_ = 8.69 ± 2.49 h
Tang et al., 2023 [59]	Chinese	Critically ill patients with nosocomial pneumonia caused by CRO	Loading dose = 100–200 mg Maintenance dose = 40–100 mg every 12 h (infusion for at least 1 h) Aerosol delivery: 25 mg/50 mg twice daily	Population PK model with the method of first-order conditional estimation-extended least square method (PMB1 and PMB2)	CL =1.56 ± 4.5 L/h; V1 = 12.5 ±5.67 L; V2 = 29.9 ± 17.3 L; C_min,ss_ = 1.9 (IQR: 1.4, 2.7) mg/L; C_max,ss_ = 5.9 (IQR: 4.6, 7.1) mg/L; AUC_ss,0–24h_ = 67.2 (IQR: 54.8, 84.2) mg·h/L
Zhang et al., 2023 [60]	Chinese	Critically ill patients with nosocomial pneumonia caused by CRO	Loading dose = 100–200 mg Maintenance dose = 40–100 mg every 12 h (infusion for at least 1 h)	Population PK model with the method of first-order conditional estimation-extended least square method (PMB1 and PMB2)	CL = 1.56 L/h; V1 = 12.5 L; V2 = 29.9 L; C_min,ss_ = 1.9 (IQR: 1.4, 2.7) mg/L; C_max,ss_ = 5.9 (IQR: 4.6, 7.1) mg/L; AUC_ss,0–24h_ = 67.2 (IQR: 54.8, 84.2) mg·h/L

AUC_ss_, steady-state area under the curve; CL: plasma clearance; C_max_: maximum concentration; C_min_: minimum concentration; C_ss, avg_: average concentration at steady state; CRKP: carbapenem-resistant K. pneumonia; CRO: carbapenem-resistant organism; CRRT, continuous renal replacement therapy; ECMO: extracorporeal membrane oxygenation; HAP: hospital-acquired pneumonia; MDR: multidrug-resistant; t_1/2_: elimination half-life; VAP: ventilator-associated pneumonia; V1: volume of central compartment distribution; V2: volume of peripheral compartment distribution; Vd: volume distribution; XDR: extensive drug resistance.
